# Cardiac Tamponade from Purulent Pericarditis due to *Cutibacterium acnes*

**DOI:** 10.1155/2018/4739830

**Published:** 2018-11-18

**Authors:** Ardalon Farhat-Sabet, Robert Hull, Dustin Thomas

**Affiliations:** ^1^Department of Internal Medicine, Brooke Army Medical Center, 3551 Roger Brooke Drive, Fort Sam Houston, TX 78234, USA; ^2^Department of Cardiology, Brooke Army Medical Center, 3551 Roger Brooke Drive, Fort Sam Houston, TX 78234, USA

## Abstract

Purulent pericarditis is a potentially fatal disease with high mortality rates if untreated. *Cutibacterium acnes* (formerly *Propionibacterium acnes*) is an anaerobic bacteria that is ubiquitous in skin flora and is commonly thought of as a culture contaminant; however, it does have pathogenic potential. We present a case of purulent pericarditis secondary to *C. acnes* leading to cardiac tamponade. Initial stabilization and diagnosis were made via pericardiocentesis; afterward the patient underwent a pericardial window. Due to a severe penicillin allergy, he was successfully treated with a 14-day course of vancomycin. To our knowledge, this represents only the third published case of purulent pericarditis with cardiac tamponade caused by *C. acnes* and the first case treated with a 14-day course of vancomycin.

## 1. Introduction

Purulent pericarditis is a rare subset of bacterial pericarditis that is characterized by gross or microscopic purulence in the pericardium and most commonly develops from direct contiguous spread of an intrathoracic infection (such as pneumonia or mediastinitis) or intracardiac source and less commonly via hematogenous spread. Prior to World War II, purulent pericarditis was a fairly common disease with an incidence as high as 1 in 254 persons. In today's age of modern antimicrobials, however, the incidence has decreased to 1 in 18,000 persons. Likewise, the spectrum of causal organisms has also changed, transitioning from an overwhelming majority of gram-positive organisms to the inclusion of gram-negative and anaerobic organisms. Despite decreasing incidence and improved antimicrobials, purulent pericarditis remains an important cause of morbidity and mortality as it is rapidly progressive and highly fatal if left untreated [1, 2]. Several known complications of purulent pericarditis are reported to include cardiac tamponade, constrictive pericarditis, left ventricular pseudoaneurysm, and aortic mycotic aneurysm formation, among others [1–3]. The severity of these complications makes a high index of suspicion imperative to allow prompt diagnosis and treatment.

## 2. Case Presentation

**Figure 1:**
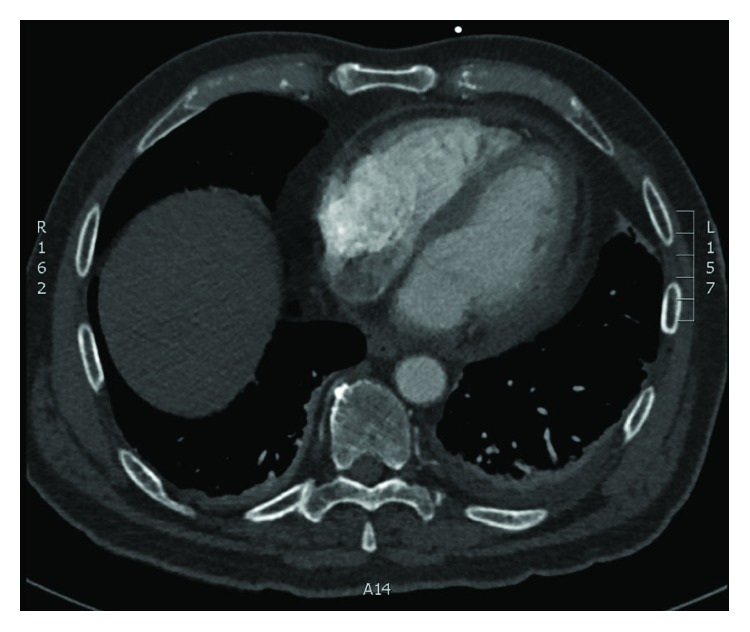
Diffuse pericardial thickening and adjacent fat stranding.

A 71-year-old male presented to cardiology clinic with a 3-week history of worsening dyspnea, night sweats, and subjective fevers. Of note, he underwent an ischemic evaluation one week prior that was significant for newly identified left pleural effusion, moderate pericardial effusion, and mild pericardial thickening on chest CT (Figure 1).

**Figure 2:**
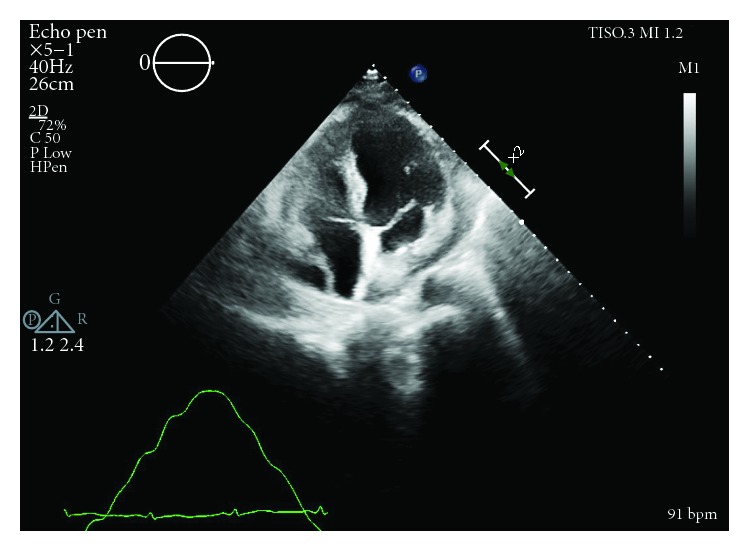
RA diastolic collapse prior to pericardiocentesis.

**Figure 3:**
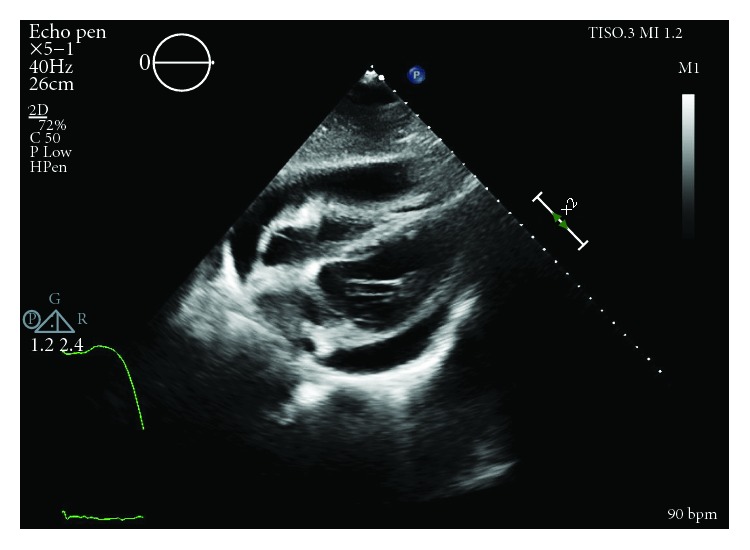
RV diastolic collapse prior to pericardiocentesis.

Initial physical exam was significant for a heart rate of 107 beats per minute and relative hypotension at 98/61 mmHg. Additionally, heart sounds were distant but jugular venous distention was absent. Later appreciated to have potential relevance, he was noted to have facial acne. Electrocardiogram revealed sinus tachycardia. Transthoracic echocardiogram (TTE) was performed which revealed that the effusion increased by greater than one centimeter since the most recent echocardiogram nine days prior and was now large and circumferential with early right ventricular diastolic collapse concerning for tamponade physiology (Figures 2 and 3). However, it also noted a nonplethoric inferior vena cava (IVC) and a lack of exaggerated respiratory inflow variability.

Urgent pericardiocentesis was performed with removal of 370 mL of nonclotting serosanguinous fluid with immediate hemodynamic and symptomatic improvement. Gram stain of pericardial fluid demonstrated microscopic purulence with mixed inflammatory cells. Empiric broad-spectrum antibiotic therapy with vancomycin was initiated. Both 72-hour aerobic cultures and anaerobic cultures held for six days were negative. Full laboratory data from before and after the pericardiocentesis can be found in the table below (Table 1).

**Figure 4:**
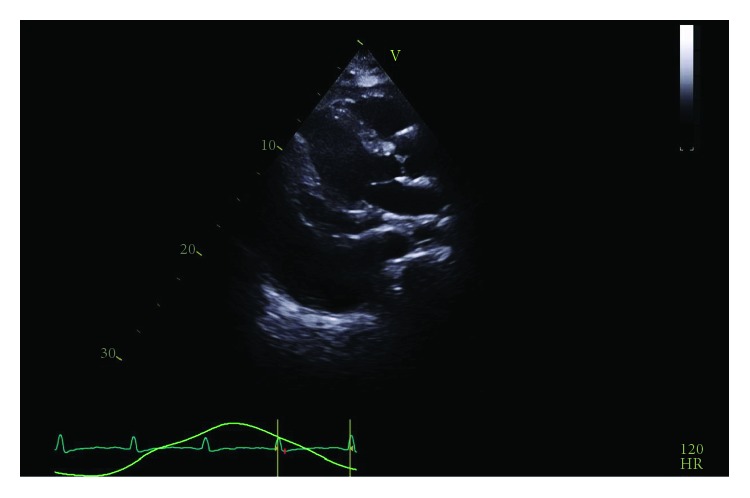
Recurrent circumferential pericardial effusion and large pleural effusion demonstrated prior to pericardial window.

Repeat bedside transthoracic echocardiogram on day three demonstrated resolution of the previously noted anterior effusion. With these imaging findings and with minimal pericardial drain output noted, the drain was removed. On day five, the patient experienced recurrent tachycardia to 121 beats per minute, relative hypotension at 97/64 mmHg, and tachypnea to 28 breaths per minute, prompting evaluation for pulsus paradoxus which was found to be 18 mmHg. Repeat TTE showed a large effusion and recurrent evidence of tamponade physiology (Figure 4). Following a heart team discussion, subxiphoid pericardial window and left-sided chest tube for pleural effusion were performed with removal of 800 mL and 200 mL of serosanguinous output, respectively. Anterior adhesions of the pericardium as well as loculated posterior pericardial effusion requiring blunt dissection were noted intraoperatively.

**Figure 5:**
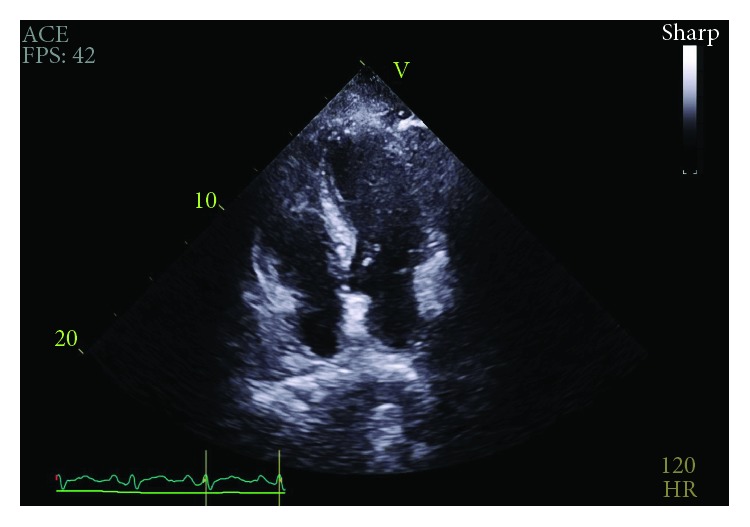
Resolution to trace pericardial effusion 8 weeks after completion of therapy.

**Figure 6:**
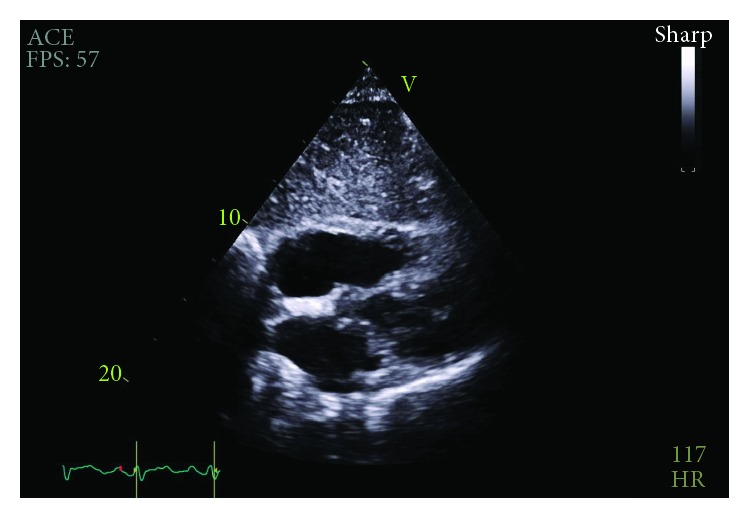
TTE performed on hospital follow-up showing no significant pericardial effusion.

Intraoperative anaerobic cultures of the pleural and pericardial fluid grew *C. acnes* after six and seven days, respectively, and anaerobic blood cultures from admission grew the same organism two weeks later. These results can be found in the table below (Table 2). Histopathology of operative pericardial samples showed fibrinous pericarditis with granulation tissue formation. Given the patient's history of anaphylaxis to penicillin, he was initiated on a 14-day course of parenteral vancomycin. The patient was discharged from the hospital in stable condition and his symptoms remained controlled at follow-up. Also noted on follow-up was resolution of the pericardial effusion as seen on TTE (Figures 5 and 6).

## 3. Discussion

Although its incidence is decreasing, purulent pericarditis remains an important clinical diagnosis to make given the high attributable morbidity and mortality rates. Classically, purulent pericarditis presents with fever, dyspnea, and tachycardia, with a median time from symptom onset to presentation of seven days. Clinicians must have a high index of suspicion as it infrequently presents with the classic features of acute pericarditis such as pericardial chest pain, electrical alternans, and a pericardial friction rub, all of which were absent in this case. Electrocardiogram findings are variable and often normal, lacking the classic diffuse ST segment elevation and PR interval depression commonly seen in idiopathic or viral pericarditis [1, 3].

It is prudent to understand the complicated interplay that can occur with the coexistence of hemodynamically significant pericardial disease and intravascularly volume-contracted states, such as that which occurs with sepsis physiology. Elevated pericardial pressure leads to compromised right ventricular filling greater than left ventricular filling resulting in impairment in stroke volume (SV). Cardiac output can be maintained in the setting of low SV with tachycardia until filling is impaired below a critical level or various compensatory mechanisms are overwhelmed as seen in classic tamponade. Thus, low preload states should be corrected with volume expansion. It is important to realize that tamponade can even occur with normal central venous pressures, so-called low-pressure tamponade [4]. As our report emphasizes, utilization of echocardiography can detect early tamponade changes that precede severe hypotension allowing for urgent, not emergent, pericardiocentesis in a more controlled setting.

Suspicion of purulent pericarditis should prompt urgent pericardiocentesis, a procedure which is both diagnostic and therapeutic. Aspirated fluid should be sent for cytology and bacterial, fungal, and tuberculous cultures. Additionally, blood cultures should be drawn to evaluate for bacteremia given the association with hematogenous spread [3]. Cultures should be kept for a minimum of seven days, as some organisms such as *Cutibacterium acnes* are unlikely to grow during the typical five-day incubation period [5, 6].

Identification and aggressive treatment of the infectious source must be initiated as soon as the diagnosis is made. Pneumonia and empyema are the most common sources, but other potential sources include mediastinitis, periodontal infection, subphrenic abscess, and sepsis [2]. Risk factors for the development of this disease include immunosuppression, chest wall trauma, and increased alcohol intake [2, 7].

Early detection and diagnosis are paramount in order to initiate appropriate antimicrobial therapy and drainage. Prompt treatment can prevent complications such as constrictive pericarditis and cardiac tamponade [3]. Treatment options for pericardial fluid drainage include percutaneous pericardiocentesis and surgical pericardiotomy. Percutaneous pericardiocentesis provides rapid drainage; however, approximately 40% of patients go on to require surgical pericardiotomy after initial drainage [3]. Surgical pericardiotomy has higher success rates and lower rates of associated constrictive pericarditis [1]. Adjunctive interventions, such as pericardiectomy or intrapericardial fibrinolysis, may be required in patients with dense adhesions, loculated and frankly purulent effusions, recurrent tamponade, persistent infection, and evidence of constrictive pericarditis [8–11].

*Cutibacterium* are gram-positive, catalase-positive, anaerobic bacilli. In general, *Cutibacterium* species have relatively low virulence and are slow-growing [12]. This pathogen is typically susceptible to penicillins, clindamycin, macrolides, quinolones, cephalosporins, and carbapenems. Resistance to aminoglycosides and metronidazole has been reported [13]. Given our patient's history of anaphylaxis with penicillin, the decision was made to avoid penicillin desensitization and parenteral vancomycin therapy was selected in consultation with infectious disease specialists. Other reported antibiotic combinations for treatment of purulent pericarditis due to *C. acnes* include intravenous penicillin G for four weeks followed by twelve weeks of oral amoxicillin/clavulanic acid and intravenous penicillin G for six weeks followed by oral doxycycline for six weeks [5, 6]. There is no widely agreed-upon treatment duration, and duration should be guided by the specific source.

*C. acnes* is a rare cause of purulent pericarditis, although the incidence may be underestimated. Given its role as a normal component of skin flora, the growth of *C. acnes* is often dismissed and attributed to culture contamination. Additionally, the longer incubation time required to grow *C. acnes* may result in under detection given that most hospital systems discard blood cultures after a five-day incubation period [5, 6]. In the setting of pathologic infection, *C. acnes* has immune-stimulatory effects that promote inflammatory cell infiltration and fibrosis. Thus, when identified as the causative pathogen in pericardial infections, it commonly leads to pericardial inflammation, pericardial effusions, and constrictive physiology [14].

## 4. Conclusion

We reported a rare case of purulent pericarditis caused by *Cutibacterium acnes*, complicated by recurrent cardiac tamponade requiring surgical pericardiotomy which was successfully treated with a 14-day course of parental vancomycin due to a penicillin allergy. This case highlights the importance of a high clinical suspicion for the diagnosis given the lack of classic exam and electrocardiogram findings of pericarditis. Additionally, our case highlights the need to communicate directly with the microbiology lab to ensure adequate incubation times to detect less common pathogens.

## Figures and Tables

**Table 1:** Laboratory results from before and after pericardiocentesis.

	Before pericardiocentesis	After pericardiocentesis	Before pericardial window	After completion of intravenous antibiotic therapy
WBC (×10^3^)	13.1 (92.5% neutrophils)	9.4 (59.2% neutrophils)	10.7 (76% neutrophils)	5.3 (59% neutrophils)
Hemoglobin (g/dL)	12.3	10.4	9.5	8.0
Hematocrit (%)	37.1	31.8	29.1	25.3
Platelets (fL)	166	356	294	220
C-reactive protein (mg/dL)	7.20	Not available	Not available	2.5
Erythrocyte sedimentation rate (mm/hr)	104	Not available	Not available	Not available

**Table 2:** Pleural and pericardial fluid analysis.

	Pericardial fluid (pericardiocentesis)	Pericardial fluid (intraoperative)	Pleural fluid (intraoperative)
WBCs	Moderate	Moderate	Few present
RBCs	Too numerous to count	Too numerous to count	Too numerous to count
Cell count	4133 nucleated cells (73% segmented neutrophils)	467 nucleated cells (56% segmented neutrophils)	489 nucleated cells (26% segmented neutrophils)
Appearance	Bloody, purulent	Turbid	Turbid
pH	Not available	7.860	7.99
Triglycerides	Not available	127	153
Amylase (U/L)	Not available	18	20
Glucose	99	116	125
LDH (IU/L)	1545	389	1513
Organisms on staining	None seen	None seen	None seen
Fungal stain	Negative	Negative	Negative
Acid-fast stain	Negative	Negative	Negative
Culture	No growth (but discarded after 72 hours)	*Cutibacterium acnes*	*Cutibacterium acnes*
